# Cross-Sectional Study on the Correlation Between Menstrual Cycle Phases and Suicidal Behavior: A Histological Analysis

**DOI:** 10.7759/cureus.81346

**Published:** 2025-03-28

**Authors:** Anamika Nath, Priyanka Jain, Prabir Hazarika

**Affiliations:** 1 Department of Forensic Medicine, Tezpur Medical College and Hospital, Tezpur, IND; 2 Department of Pathology, Tezpur Medical College and Hospital, Tezpur, IND

**Keywords:** cadaver, menstrual cycle, premenstrual syndrome, reproductive age group, suicide

## Abstract

Background

Suicide remains a significant global public health issue. The influence of menstrual cycle phases on mental health, especially in conditions like premenstrual dysphoric disorder (PMDD), has raised concerns about possible connections between hormonal changes and suicidal tendencies. Previous research has produced mixed findings regarding the relationship between menstrual phases and suicide. This study aims to investigate this potential correlation through histopathological examination.

Methods

This cross-sectional comparative study was conducted at a government medical college in Northeastern India, involving 50 women who died by suicide, aged 12-50 years. Histopathological examination of the uterus was used to determine the menstrual cycle phase of the deceased. Data collection was performed using Microsoft Excel (Microsoft Corporation, Redmond, Washington, United States), and statistical analysis was conducted with IBM SPSS Statistics for Windows, Version 21.0 (Released 2012; IBM Corp., Armonk, New York, United States).

Results

Among the 50 suicide cases, the early proliferative phase accounted for 18 cases (36%), followed by the late proliferative phase with 14 cases (28%), the late secretory phase with eight cases (16%), the menstrual phase with four cases (8%), pregnancy with four cases (8%), and the early secretory phase with two cases (4%). Hanging was the predominant method of suicide, accounting for 32 cases (64%). The majority of suicide victims (n=22; 44%) were between the ages of 16 and 20. Statistical analysis indicated no significant association between menstrual phase and method of suicide (p=0.460) or between age group and menstrual phase (p=0.243). However, the distribution of suicide cases across menstrual phases showed statistical significance (p<0.001), with an overrepresentation in the proliferative phases.

Conclusion

The findings revealed that the distribution of suicide cases across menstrual phases is not random, with a statistically significant overrepresentation in the proliferative phases. The choice of suicide method does not appear to be significantly associated with the menstrual phase, suggesting that other factors influence method selection. However, due to the small sample size, the detection of smaller but clinically relevant associations may be limited.

## Introduction

Suicide remains a major global health crisis, claiming over 800,000 lives annually and ranking among the leading causes of death worldwide [1]. The World Health Organization (WHO) highlights that suicide rates are particularly high in low- and middle-income countries, accounting for more than 77% of global suicides [2]. Suicide remains a major public health issue. In India, data from the National Crime Records Bureau (NCRB) indicate a steady and alarming increase in suicide rates, rising from 9.9 per lakh population in 2017 to 12.4 per lakh population in 2022 [3]. While men are more likely to complete suicide, women tend to attempt suicide more frequently, revealing a gender-based disparity in suicidal behaviors [4].

Among women, hormonal fluctuations are increasingly being studied for their impact on mental health, particularly across different phases of the menstrual cycle. Premenstrual dysphoric disorder (PMDD), a severe form of premenstrual syndrome (PMS), affects an estimated 3-8% of reproductive-age women and is marked by severe mood disturbances, including depression, irritability, and suicidal ideation [5]. Some studies indicate that PMDD may worsen psychiatric conditions, elevating suicide risk during particular menstrual phases [6]. However, conflicting research findings make it necessary to explore this relationship further, like interactions between the immune and neuroendocrine systems [7].

The aim of this research was to investigate the correlation between menstrual cycle phases and suicidal behavior among women of reproductive age through histopathological examination of the uterus post-mortem and to cross-analyze age groups and methods of suicide with menstrual cycle phases.

## Materials and methods

Study setting and duration

This study was conducted at the Department of Forensic Medicine and Department of Pathology of Tezpur Medical College and Hospital in Tezpur, India, over a period of six months from July 1, 2024, to December 31, 2024.

Study design

A cross-sectional comparative study was undertaken to analyze the correlation between menstrual cycle phases and suicidal behavior in women of reproductive age using histopathological examination.

Sampling method

We used a convenient sampling method to collect all the cases that came for autopsy and fulfilled the inclusion criteria within our study period.

Study population

The study included 50 female cadavers aged 12-50 years who had died due to suicide.

Inclusion criteria

(a) Female cadavers aged 12-50 years where history and findings suggested the manner of death to be suicide were included in the study.

Exclusion criteria

(a) Female cadavers with other manner of death, (b) decomposed bodies, (c) suicide cases of women with known pregnancy, (d) deaths occurring more than 24 hours after a suicide attempt, (e) cases with undetermined causes of death, and (f) individuals with known hermaphroditic conditions were excluded from the study.

Ethical considerations

Ethical approval was obtained from the Institutional Human Ethical Committee of Tezpur Medical College and Hospital (approval number: 2024/081/TMC&H; date: 22/06/2024). Informed consent for the use of cadaveric samples was taken from legal guardians or next of kin in accordance with ethical guidelines. The research was done according to Helsinki guidelines and guidelines on Good Clinical Practice.

Data collection and histopathological analysis

At the time of autopsy, uterus specimens were collected, preserved in 10% buffered formalin, and labeled with age and methods of suicide. Histopathological tissue processing was done by following the steps of dehydration clearing, infiltration, and embedding. Histopathological tissue processing was done using Thermo Scientific STP120-3 automated tissue processor (Waltham, Massachusetts, United States). Dehydration was done using acetone, and as a clearing agent, xylene was used. Infiltration was done in the processor by use of paraffin, and embedding was done manually using Leuckhardt's L-moulds. Thereafter, sections of 4-5 µm thickness were made, stained with routine hematoxylin and eosin stain, and mounted with dibutylphthalate polystyrene xylene. Then histopathological examination was performed to determine the menstrual cycle phase. The endometrium was categorized into menstrual, early proliferative, late proliferative, early secretory, and late secretory phases based on standard histological criteria.

Statistical analysis

Data were recorded in Microsoft Excel (Microsoft Corporation, Redmond, Washington, United States) and analyzed using IBM SPSS Statistics for Windows, Version 21.0 (Released 2012; IBM Corp., Armonk, New York, United States) [8,9]. Statistical tests included the following: (a) chi-squared test for categorical data, (b) odds ratio for assessing risk association, (c) t-test for comparing continuous variables, and (d) logistic regression analysis to determine potential predictors of suicide. A p-value of <0.05 was considered statistically significant.

## Results

The 50 cases of suicide in female cadavers were analyzed according to different menstrual cycle phases, methods of suicide, and age groups. In the distribution of cases according to the menstrual phase, the majority of suicide cases (n=32; 64%) occurred during the proliferative phase (early and late combined), with only two cases (4%) during the early secretory phase. In four cases (8%), pregnancy was an incidental finding without any known history, as depicted in Table 1. 

**Table 1:** Distribution of cases based on menstrual cycle phases

Menstrual phase	Number of cases	Percentage
Early proliferative	18	36%
Late proliferative	14	28%
Late secretory	8	16%
Menstrual	4	8%
Pregnancy	4	8%
Early secretory	2	4%
Total	50	100%

In method preference, hanging was the predominant method of suicide (n=32; 64%), followed by poisoning (n=12; 24%), as shown in Table 2.

**Table 2:** Distribution of cases based on method of suicide

Method of suicide	Number of cases	Percentage
Hanging	32	64%
Poisoning	12	24%
Burns	4	8%
Drowning	2	4%
Total	50	100%

In age pattern, most suicide victims (n=40; 80%) were under 25 years of age, with the highest concentration (n=22; 44%) in the 16-20 age group, as shown in Table 3. 

**Table 3:** Distribution of cases based on age group

Age group	Number of cases	Percentage
12-15 years	8	16%
16-20 years	22	44%
21-25 years	10	20%
26-30 years	2	4%
31-35 years	6	12%
36-40 years	2	4%
Total	50	100%

In a cross-analysis of methods of suicide by phase of the menstrual cycle, hanging was most common during the early proliferative phase (n=14; 28%), while all drowning cases occurred during the late proliferative phase (Table 4).

**Table 4:** Cross-analysis of methods of suicide by phase of the menstrual cycle

Menstrual phase	Hanging	Poisoning	Burns	Drowning	Total
Early proliferative	14	2	2	0	18
Late proliferative	8	4	0	2	14
Early secretory	0	2	0	0	2
Late secretory	4	2	2	0	8
Menstrual	2	2	0	0	4
Pregnancy	4	0	0	0	4
Total	32	12	4	2	50

In the age and menstrual phase correlation, the early proliferative phase showed the highest incidence among the 16-20-year age group (n=10; 20%) (Table 5).

**Table 5:** Cross-analysis of age group and menstrual phases

Age group	Early proliferative	Late proliferative	Early secretory	Late secretory	Menstrual	Pregnancy	Total
10-15 years	4	2	0	0	2	0	8
16-20 years	10	6	0	4	0	2	22
21-25 years	2	4	0	2	0	2	10
26-30 years	0	0	0	2	0	0	2
31-35 years	2	2	0	0	2	0	6
36-40 years	0	0	2	0	0	0	2
Total	18	14	2	8	4	4	50

We investigated whether suicide cases are randomly distributed across different phases of the menstrual cycle or if some phases show an unusually high number of cases. To do this, we used a chi-squared statistical test, which helped to determine if the observed distribution of suicide cases differed significantly from what would be expected by random chance. The chi-squared test produced a value of 24.42, with 5 degrees of freedom, the critical value at a significance level of 0.05 was 11.07, and the p-value was less than 0.001 (Table 6).

**Table 6:** Chi-squared test for menstrual phase distribution Chi-squared value: 24.42; df: 5; critical value at p=0.05; df=5 is 11.07; p<0.001 P<0.001 is considered statistically significant. O: observed; E: expected; df: degree of freedom

Menstrual phase	O	E	(O-E)²	(O-E)²/E
Early proliferative	18	8.33	93.52	11.22
Late proliferative	14	8.33	32.19	3.86
Early secretory	2	8.33	40.19	4.82
Late secretory	8	8.33	0.01	0.001
Menstrual	4	8.33	18.85	2.26
Pregnancy	4	8.33	18.85	2.26
Total	50	50		24.42

We investigated whether there's a statistically significant relationship between a person's menstrual phase and their chosen method of suicide. We used a chi-squared test to investigate this potential connection. The chi-squared test produced a value of 14.88, with 15 degrees of freedom, the critical value at a significance level of 0.05 was 25.00, and the p-value was less than 0.460. This result indicates no statistically significant association between the menstrual phase and the method of suicide. This suggests that the method choice is independent of the menstrual phase. We also explored whether the age distribution of individuals who died by suicide differs significantly across various menstrual phases and used a chi-squared statistical test to examine this potential relationship. The chi-squared test produced a value of 29.51, with 25 degrees of freedom, the critical value at a significance level of 0.05 was 37.65, and the p-value was less than 0.243. This result indicates no statistically significant association between age group and menstrual phase in suicide cases, though this could be affected by the small sample size in some cells. We analyzed the odds ratio of suicide in proliferative phases (early and late combined) vs. other phases and found the odds ratio as follows: (32/18)/(15/35)=4.15. This means women in the proliferative phase have approximately 4.15 times higher odds of suicide compared to women in other menstrual phases, while the 95% confidence interval was 1.84-9.37. As the confidence interval does not include 1, this odds ratio is statistically significant. A logistic regression model predicting the likelihood of specific suicide methods based on menstrual phase and age showed the proliferative phase is a significant predictor of hanging as a suicide method (p<0.05), while age shows some influence but doesn't reach statistical significance at the conventional p<0.05 level (Table 7).

**Table 7:** Logistic regression for hanging as a suicide method with menstrual phase and age

Predictor	Odds ratio	P-value
Proliferative phase	2.74	0.042
Age (16-20)	1.92	0.089
Age (21-30)	1.16	0.384

The age group 21-30 is significantly associated with poisoning as a suicide method (p<0.05), while the proliferative phase does not appear to significantly influence the choice of poisoning (Table 8).

**Table 8:** Logistic regression for poisoning as a suicide method with menstrual phase and age

Predictor	Odds ratio	P-value
Proliferative phase	0.57	0.148
Age (16-20)	1.28	0.317
Age (21-30)	2.03	0.046

Using the point-biserial correlation analysis for age group and methods of suicide, we found that there is a significant negative correlation between age and hanging (younger women more likely to use this method) and there is a significant positive correlation between age and poisoning (older women more likely to use this method) (Table 9).

**Table 9:** Point-biserial correlation analysis for age group and method of suicide

Method	Correlation with age	P-value
Hanging	-0.31	0.028
Poisoning	0.29	0.041
Burns	0.11	0.445
Drowning	-0.07	0.628

## Discussion

Research examining the link between menstrual cycles and suicide has yielded mixed findings. Some studies, such as by Pinkerton et al. [6], found an increased risk of suicide attempts during the premenstrual and menstrual phases, where hormone levels are at their lowest. Also, researchers have found that there are a number of medical conditions that seem to be exacerbated according to menstrual cycle phases. Though the etiology is not clear, however, research suggests intricate interactions between the immune and neuroendocrine systems [7]. Conversely, Vanezis [10] found no significant correlation between menstrual phases and suicide rates, aligning with the findings of the present study.

**Figure 1:**
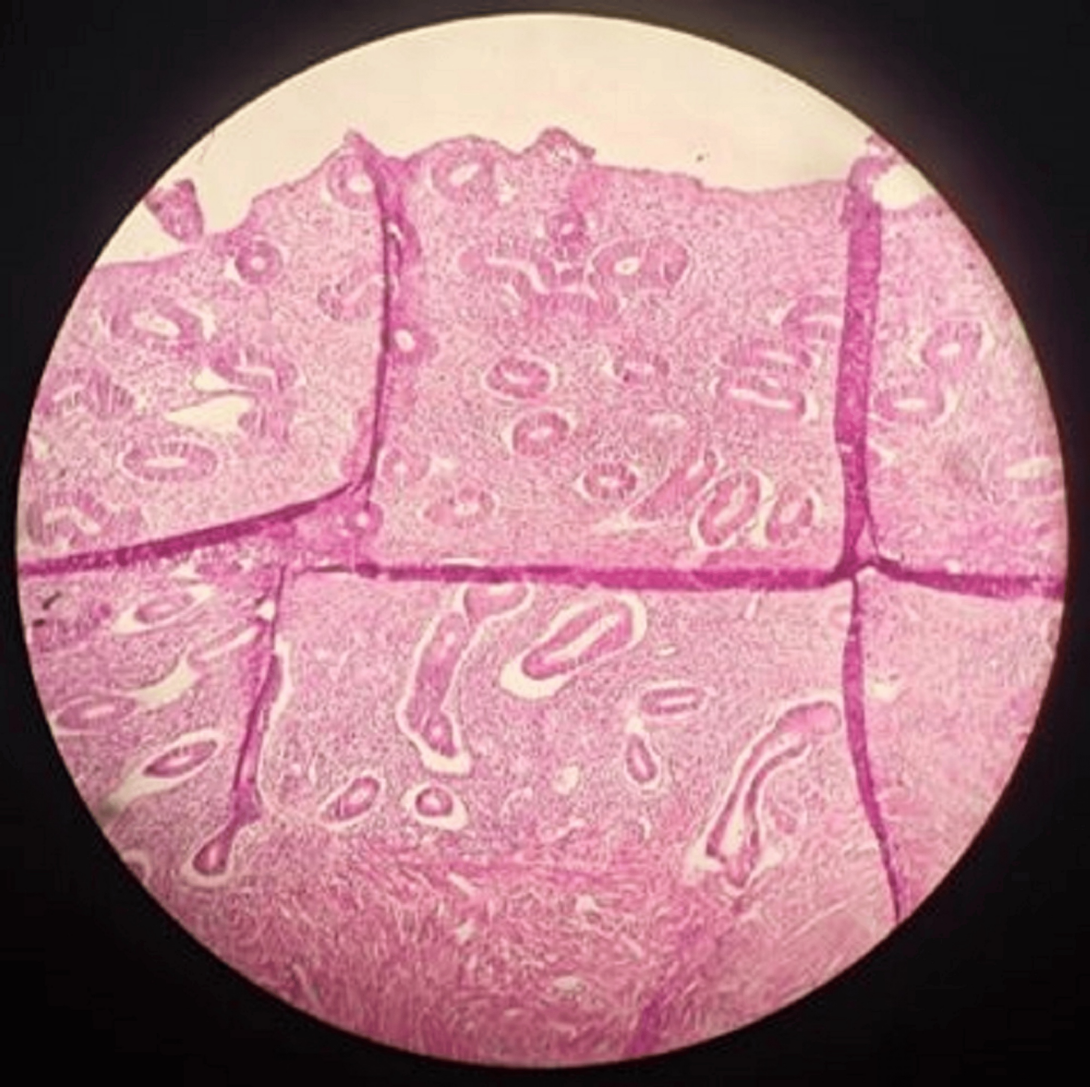
Early proliferative phase with compact glands

**Figure 2:**
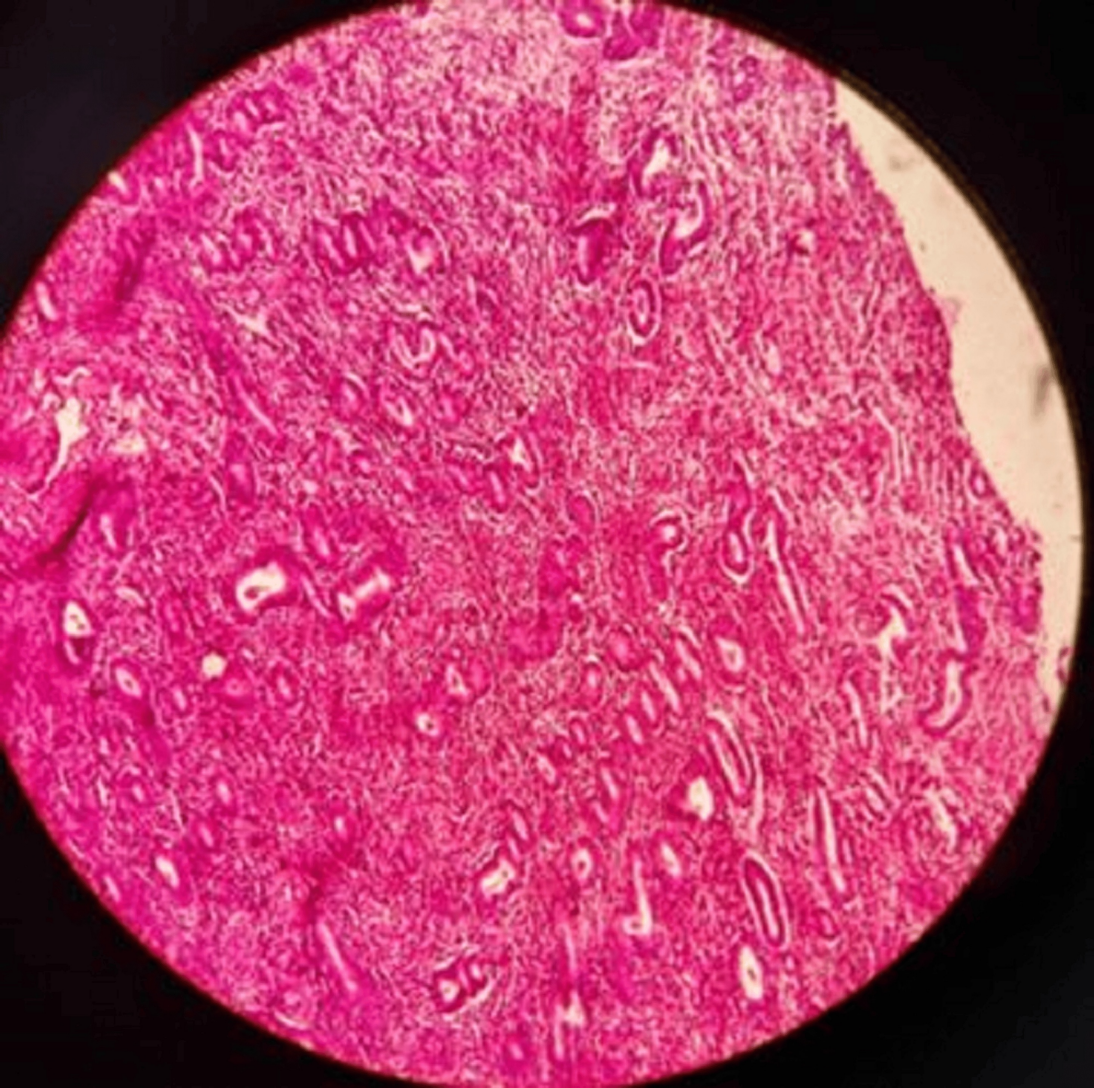
Late proliferative phase with elongated curved packed glands

The results of our study reveal several important findings. The significant distribution of suicide cases across menstrual phases (p<0.001) indicates that suicides are not randomly distributed throughout the menstrual cycle. The overrepresentation in the proliferative phases (early proliferative: n=18 (36%), as shown in Figure 1; late proliferative: n=14 (28%), as shown in Figure 2) suggests a potential relationship between these phases and suicide risk. 

**Figure 3:**
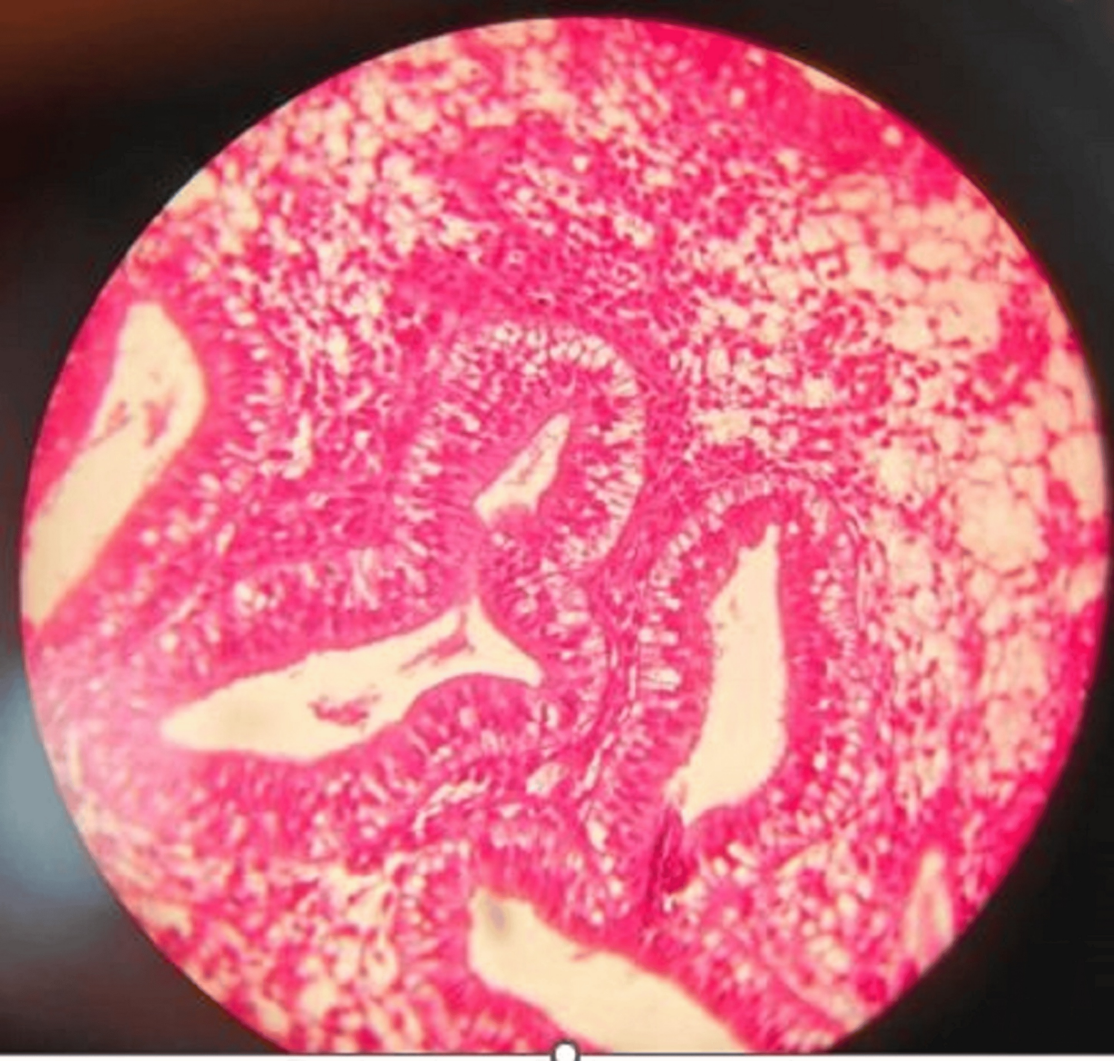
Early secretory phase with subnuclear vacuoles

**Figure 4:**
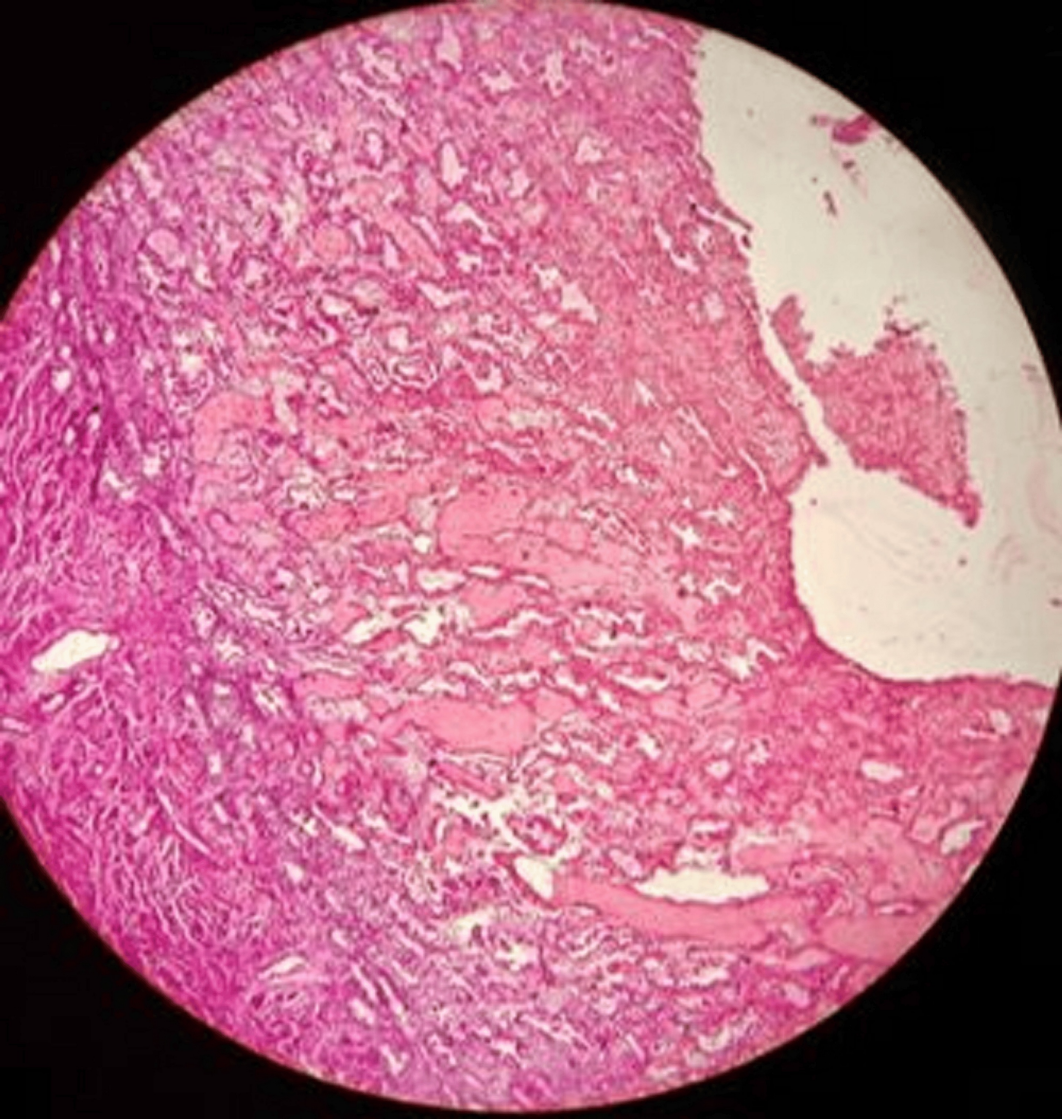
Late secretory endometrium with predecidual changes

**Figure 5:**
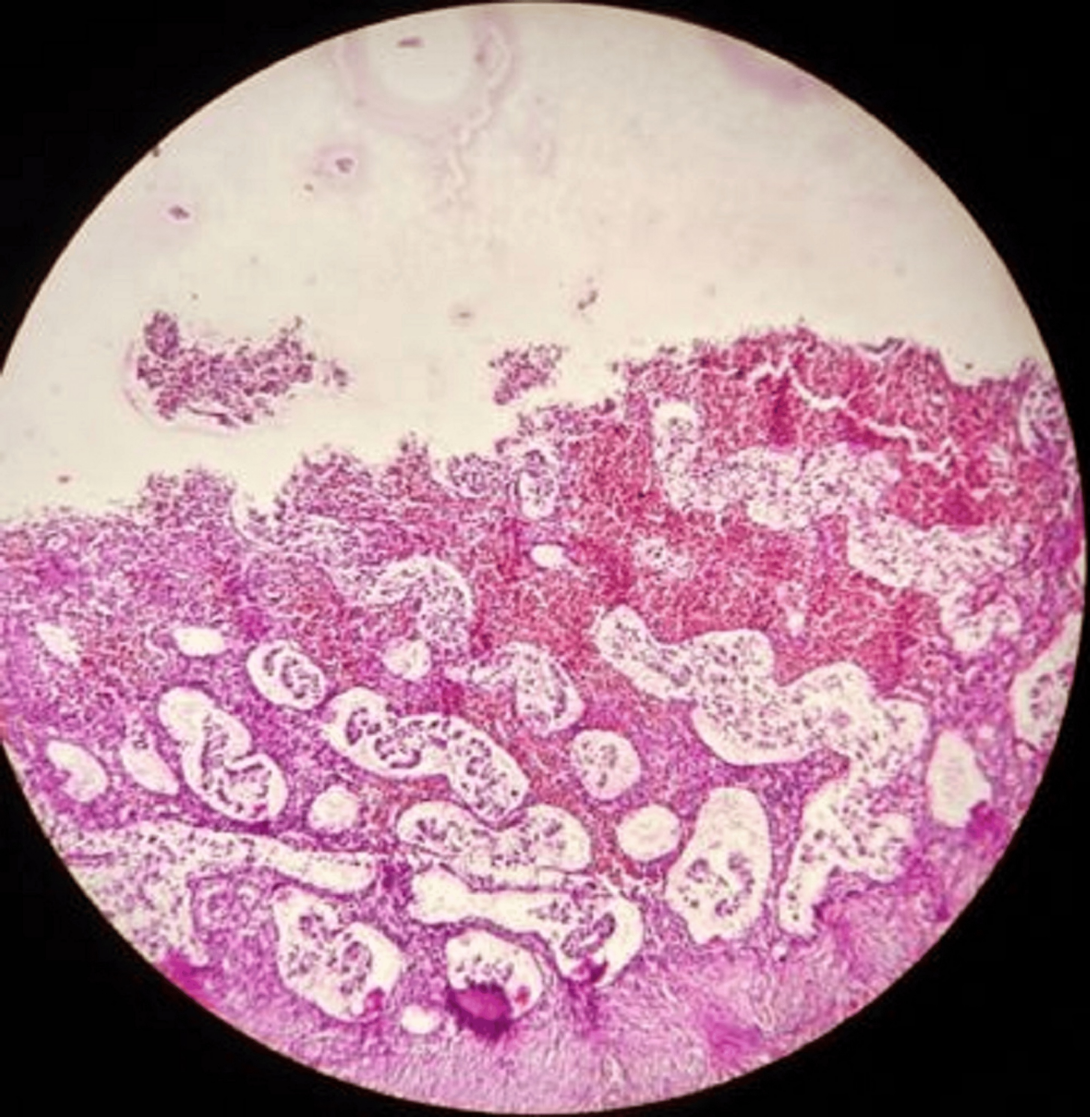
Menstruating endometrium showing shedding and hemorrhage

**Figure 6:**
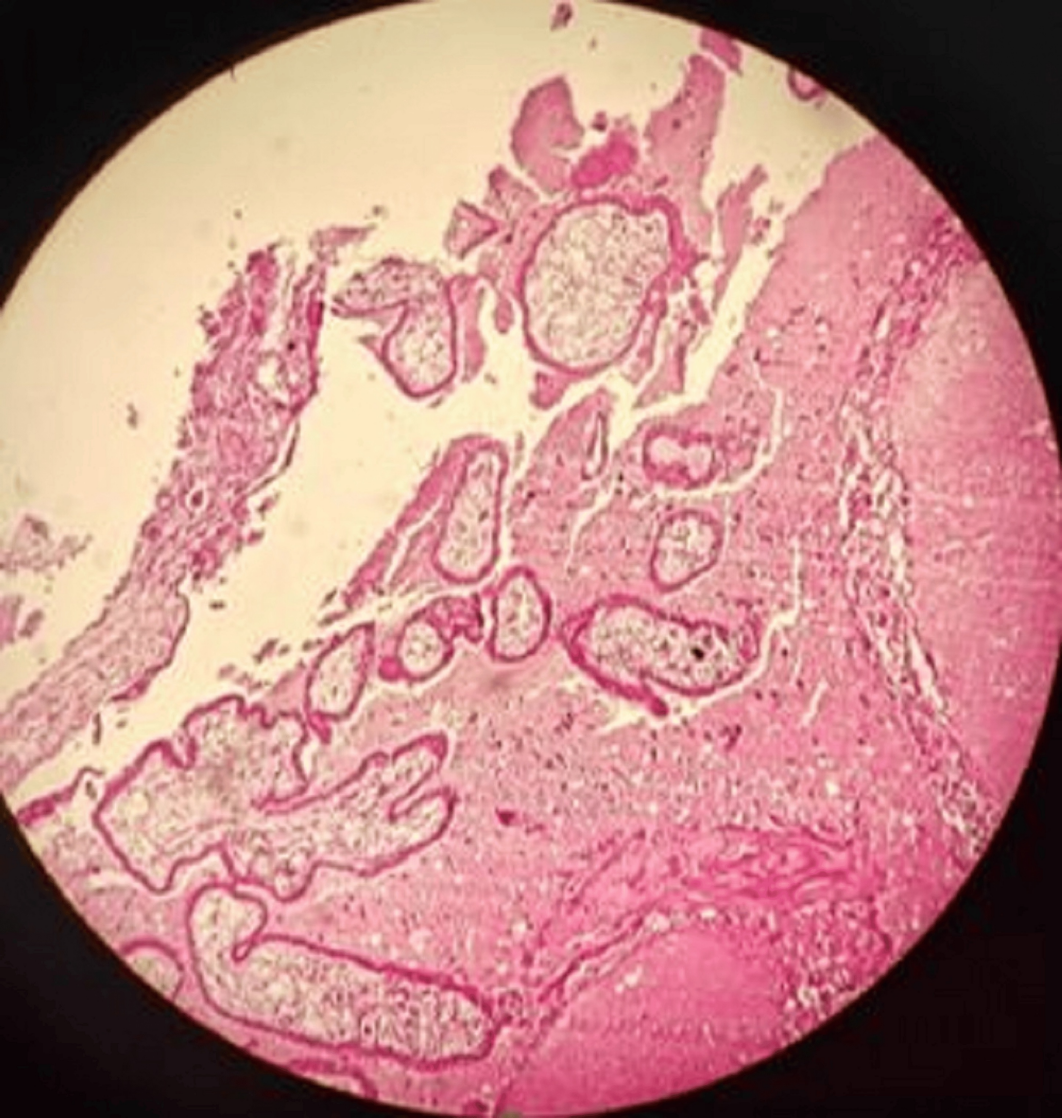
Pregnancy in the uterus showing chorionic villi

Women in the proliferative phase appear to have over four times higher odds of suicide compared to women in other phases (odds ratio=4.15; 95% CI: 1.84-9.37). This finding is particularly notable as it contradicts some previous research suggesting higher risks during the premenstrual or menstrual phases. However, we found in two cases (4%) early secretory changes, as shown in Figure 3, and in eight cases (16%) late secretory changes, as shown in Figure 4. In four cases each (8%), we found menstrual phase, as shown in Figure 5, and incidental pregnancy, as shown in Figure 6.

The proportion of suicide cases during the early proliferative phase (n=18; 36%) was significantly higher than would be expected based on the duration of this phase in a typical menstrual cycle (p=0.004). This suggests that hormonal fluctuations during this phase might influence suicide risk, possibly due to changes in estrogen levels affecting mood regulation pathways in the brain.

Regarding suicide methods, our analysis found that hanging was the predominant method (n=32; 64%), followed by poisoning (n=12; 24%), burns (n=4; 8%), and drowning (n=2; 4%). The logistic regression analysis revealed that the proliferative phase was a significant predictor of hanging as a suicide method (odds ratio=2.74; p=0.042). This could indicate that hormonal states during this phase might influence method selection, possibly through effects on impulsivity or decision-making processes.

The age distribution findings show that the majority of suicide victims (n=32; 64%) were under 25 years of age, with the highest concentration (n=22; 44%) in the 16-20 age group. The early proliferative phase showed the highest incidence among 16-20-year-olds (10 cases). However, our statistical analysis found no significant association between age group and menstrual phase in suicide cases (p=0.243), suggesting that age and menstrual phase may be independent risk factors.

Interestingly, we found significant correlations between age and method choice, with younger women more likely to use hanging (correlation=-0.31; p=0.028) and older women more likely to use poisoning (correlation=0.29; p=0.041). This age-method relationship appears to be independent of the menstrual phase, as we found no significant association between the menstrual phase and the method of suicide (p=0.460).

Menstruation is a vital physiological process that influences multiple aspects of physical and mental health. It involves complex hormonal changes that can significantly affect mood, behavior, and emotional stability. Several studies suggest that hormonal fluctuations across the menstrual cycle contribute to mood disorders, with some phases posing a higher risk for suicidal ideation and attempts.

PMDD is a severe form of PMS that affects approximately 3-8% of menstruating women and is characterized by extreme mood disturbances, depression, anxiety, irritability, and suicidal thoughts [11]. Women with PMDD often experience symptoms severe enough to disrupt daily life and may be more vulnerable to self-harm during the late luteal and menstrual phases [12]. Studies suggest that lower levels of estrogen and progesterone during these phases may contribute to increased suicide risk [13].

A study by Martin et al. found a higher incidence of suicide attempts in the follicular phase, suggesting that individual hormonal responses might vary [14]. Similarly, research conducted by Smith et al. [15] indicated a marginal increase in suicidal behavior during the late luteal phase but lacked conclusive statistical significance.

Hormonal influences on mood disorders are well documented, and some researchers argue that suicidal behavior in women could be more closely related to underlying psychiatric conditions than the menstrual phase alone. Studies by Pearlstein et al. [16] and Wikman et al. [17] indicate that women with PMDD and pre-existing depressive disorders are at a heightened risk for suicidal ideation, suggesting that mental health interventions should be tailored to menstrual health concerns.

The relatively small sample size, particularly when stratified across multiple categories, may limit the detection of smaller but clinically relevant associations. The chi-squared test results should be interpreted with caution due to small cell sizes in some categories.

The research was limited by its small sample size, retrospective methodology, unaccounted confounding factors, reliance on histopathological phase determination without hormonal validation, and single-center design. To advance understanding in this area, we recommend conducting larger multi-center studies, incorporating hormonal assessments alongside histopathological findings, integrating comprehensive psychiatric evaluations, developing public health initiatives focused on menstrual health's psychological effects, and implementing routine mental health screenings for reproductive-age women, particularly those with PMDD or depression.

## Conclusions

This study contributes to the ongoing discussion about the relationship between menstrual cycles and suicide. While no statistically significant correlation was found between specific menstrual phases and suicide methods or age groups, we did observe a significant non-random distribution of suicide cases across menstrual phases, with overrepresentation in the proliferative phases. The findings highlight the need for more in-depth research. Given the role of PMDD and other mental health disorders in suicidal behavior, future studies should incorporate hormonal analysis and psychiatric evaluation to provide a clearer understanding of suicide risk among menstruating women.
